# Platelet-derived growth factor signaling modulates adult hair follicle dermal stem cell maintenance and self-renewal

**DOI:** 10.1038/s41536-017-0013-4

**Published:** 2017-04-14

**Authors:** Raquel González, Garrett Moffatt, Andrew Hagner, Sarthak Sinha, Wisoo Shin, Waleed Rahmani, Andrew Chojnacki, Jeff Biernaskie

**Affiliations:** 1 Department of Comparative Biology and Experimental Medicine, Faculty of Veterinary Medicine, University of Calgary, Calgary, Alberta T2N 4N1 Canada; 2 Department of Surgery, Faculty of Medicine, University of Calgary, Calgary, Alberta T2N 4N1 Canada; 3 Alberta Children’s Hospital Research Institute, University of Calgary, Calgary, Alberta T2N 4N1 Canada; 4 Hotchkiss Brain Institute, University of Calgary, Calgary, Alberta T2N 4N1 Canada

## Abstract

Hair follicle regeneration is dependent on reciprocal signaling between epithelial cells and underlying mesenchymal cells within the dermal papilla. Hair follicle dermal stem cells reside within the hair follicle mesenchyme, self-renew in vivo, and function to repopulate the dermal papilla and regenerate the connective tissue sheath with each hair cycle. The identity and temporal pattern of signals that regulate hair follicle dermal stem cell function are not known. Here, we show that platelet-derived growth factor signaling is crucial for hair follicle dermal stem cell function and platelet-derived growth factor deficiency results in a progressive depletion of the hair follicle dermal stem cell pool and their progeny. Using *αSMACreER* ^*T2*^ *:Rosa* ^*YFP*^ *:Pdgfrα* ^*flox*^ mice, we ablated *Pdgfrα* specifically within the adult hair follicle dermal stem cell lineage. This led to significant loss of hair follicle dermal stem cell progeny in connective tissue sheath and dermal papilla of individual follicles, and a progressive reduction in total number of anagen hair follicles containing YFP^+ve^ cells. As well, over successive hair cycles, fewer hair follicle dermal stem cells were retained within each telogen hair follicle suggesting an impact on hair follicle dermal stem cell self-renewal. To further assess this, we grew prospectively isolated hair follicle dermal stem cells (Sox2GFP^+ve^ αSMAdsRed^+ve^) in the presence or absence of platelet-derived growth factor ligands. Platelet-derived growth factor-BB enhanced proliferation, increased the frequency of Sox2^+ve^ hair follicle dermal stem cell progeny and improved inductive capacity of hair follicle dermal stem cells in an ex vivo hair follicle formation assay. Similar effects on proliferation were observed in adult human SKPs. Our findings impart novel insights into the signals that comprise the adult hair follicle dermal stem cell niche and suggest that platelet-derived growth factor signaling promotes self renewal, is essential to maintain the hair follicle dermal stem cell pool and ultimately their regenerative capacity within the hair follicle.

## Hair loss: The protein that signals regeneration

Signals from a protein that regulates cell division are essential to maintain the stem cells that regenerate hair follicles. Jeff Biernaskie and colleagues at Canada’s University of Calgary found signals from platelet-derived growth factor (PDGF) promote self-renewal of ‘hair follicle dermal stem cells’ (hfDSCs)—cells present at the bottom of hair follicles important for their regeneration. They ‘turned off’ the gene responsible for PDGF production in hfDSCs in mice. This led to a significant reduction in the stem cells with successive hair cycles. They also tested the effects of PDGF signaling molecules on isolated hfDSCs and found they improved their ability to proliferate and to induce follicle regeneration. The results suggest disruption to PDGF signaling may contribute to hair loss. PDGF could be an important additive to rapidly expand hfDSCs *ex vivo* for cell-based therapies.

## Introduction

The hair follicle (HF) is a unique model system of adult tissue regeneration. To initiate the regenerative cycle, stem cells residing in the epithelial bulge and secondary germinal zone are activated by signals emanating from specialized mesenchymal cells that comprise the dermal papilla (DP). Indeed, the DP is required for successful HF regeneration,^1, 2^ and consequently, alopecia is often associated with cell death or atrophy of the instructive cells comprising the DP.

Recent work has identified the existence of a self-renewing mesenchymal stem cell that resides within the adult HF and functions to regenerate the connective tissue sheath (CTS) and contribute new cells to the DP with each new hair cycle.^3^ Genetic depletion of these HF dermal sheath stem cells (hfDSCs) resulted in impaired hair growth and prevented conversion to a larger hair type,^3^ a process that requires an increase in DP cells,^4^ indicating their importance in repopulating these dermal lineages within the HF and specifically in maintaining sufficient numbers of DP cells to enable inductive competency throughout life. hfDSCs are α-smooth muscle actin and Sox2-expressing cells that reside specifically within the HF dermal cup (DC) that surrounds the HF bulb. When prospectively isolated and grown in vitro, they are highly enriched for self-renewing, colony forming cells that have previously been referred to as skin-derived precursors (“SKPs”).^5–7^ SKPs are non-adherent colony forming cells that proliferate in response to basic fibroblast growth factor (bFGF) from cultures of dissociated dermis. SKPs are thought to be the in vitro derivative of hfDSCs^3^ as, like hfDSCs, transplanted SKPs are able to induce *de novo* HF formation or reconstitute the DP and CTS of existing follicles and subsequently modify the type of hair that was produced^5^ thereby distinguishing them from other fibroblast populations within the skin and also highlighting their significant therapeutic potential.

Based on this, we propose that hfDSC dysfunction may contribute to the pathogenesis underlying hair loss and paradoxically, healthy hfDSCs may serve as a novel cell replacement strategy to rejuvenate the inductive mesenchyme and ultimately restore HF function in disease/injured or aged skin. In either case, translation toward clinical therapy will require a thorough understanding of the molecular signals that modulate hfDSC self-renewal and fate choice within the mesenchymal lineage.

Here, we have examined the role of platelet-derived growth factor (PDGF) signaling within the adult hfDSC niche. Previous work has shown that isolated dermal fibroblasts exhibit enhanced proliferation and migration in the presence of PDGF ligands.^8–10^ After skin injury, application of PDGF accelerates the rate of skin wound closure,^11–13^ and as such, has been utilized clinically for treatment of ulcerative wounds.^14–16^ Conventional *Pdgfrα* null mice exhibit robust skin defects including dermal hypoplasia.^17^ However, more recent work employing mice with a conditional deletion of both *Pdgfrα* and *Pdgfrβ* within the developing DP of embryonic HFs, reported that PDGF signaling in dermis is not required for normal HF morphogenesis.^18^ Previous studies using global deletion of PDGF-A ligand similarly found little effect on initial HF formation.^19^ Intriguingly though, both studies reported robust postnatal dermal thinning, loss of follicular mesenchyme, and an inability to initiate anagen HF regeneration.

Based on these reports and our previous characterization of hfDSCs, we hypothesized that PDGF signaling may be an important regulator of adult hfDSC function. If the ability of hfDSCs to populate the DP and/or CTS compartments at the onset of anagen was compromised, this might account for the observed defects in HF regeneration. We found that conditional genetic deletion of *Pdgfrα* in hfDSCs resulted in a significant decline in hfDSC progeny, specifically within the CTS compartment. Exposure of prospectively isolated hfDSCs to PDGF ligands increased proliferation as well as their capacity for serial (clonal) colony formation and enhanced their capacity to induce new HF formation. Importantly, exposure to PDGF also enhanced proliferation of adult human SKPs (which are the in vitro derivative of hfDSCs). Taken together, our findings suggest that PDGF is critical for maintaining normal hfDSC function within the adult skin.

## Results

### Conditional *Pdgfrα* deficiency in hfDSCs causes progressive loss of progeny and depletion of the stem cell pool

We generated *αSMACreER* ^*T2*^ *:Rosa* ^*YFP*^ *:Pdgfrα* ^*flox/flox*^ mice to perform in vivo fate mapping experiments of adult hfDSCs and their PDGF signaling deficient progeny. Recombination and successful excision was confirmed by qPCR (Supplementary Fig. 1C–E). No obvious hair phenotype was observed in the *Pdgfrα* ^*flox/flox*^ animals in comparison to their *Pdgfrα* ^*+/+*^ littermates. Tamoxifen administration (P21-P23-P25) did not affect the onset of either second or third adult anagen in any genotype. In addition, tamoxifen administration did not affect body weight (data not shown).

**Fig. 1:**
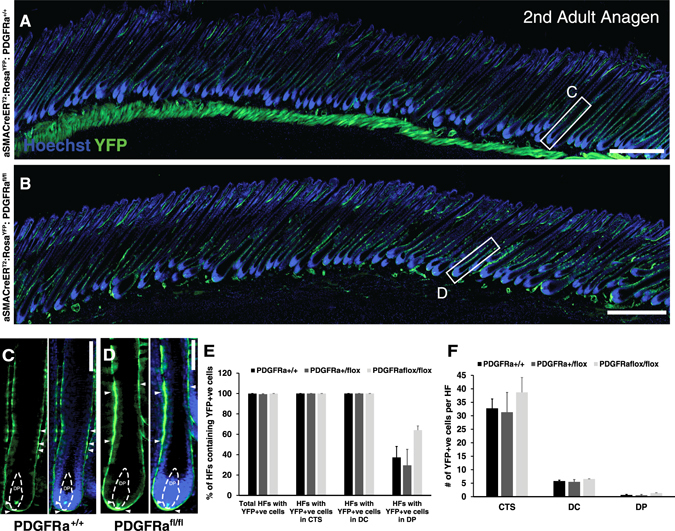
*In vivo* fate mapping of *Pdgfrα-*deficient hfDSCs during second adult anagen. **a** Skin from αSMACreER^T2^ *:*Rosa^YFP^ *:Pdgfrα* ^*+/+*^ or **b** *Pdgfrα* ^*flox/flox*^ animals after Cre-induced recombination. The backs of the animals were depilated at P21 (telogen) and tamoxifen was administered by gavage at P21, P23, and P25 (1 mg/day/animal). The subsequent anagen phase was documented and compared between tamoxifen-treated wild-type (*n* = 3), heterozygous (*n* = 4), or *Pdgfra* ^*flox/flox*^ (*n* = 3), carrying Cre animals. Skin was harvested during second anagen (P33-P35). **c** Magnified view of an individual HF from *Pdgfrα* ^*+/+*^ skin (*white box* in (**a**)) showing YFP^+ve^ cells (*green, arrowheads*) in each of the dermal compartments of the HF, i.e., CTS, DC, and DP outlined by *dashed lines*. **d** Magnification of a HF from *Pdgfrα* ^*flox/flox*^ skin (*white box* in (**b**)) for comparison. **e** Percentage of HFs containing YFP^+ve^ cells. Total number of YFP^+ve^ HFs per genotype are represented, as well as the percentages of HFs with YFP^+ve^ cells present in each dermal compartment. **f** Mean number of YFP^+ve^ cells per HF present in each component of the dermal compartment in *Pdgfrα* ^*+/+*^ *; Pdgfr* ^*+/flox*^ and *Pdgfrα* ^*flox/flox*^ animals. Data are mean ± SEM (*n* = 10 animals). Kruskal–Wallis test (*p* = n.s.). Scale bar represents 500 (**a**, **b**) or 50 µm (**c**, **d**). See also Fig. S1

At second anagen (~P32), nearly all HFs contained YFP^+ve^ cells, regardless of the genotype (Fig. 1a–d). The percentage of total YFP^+ve^ HFs was similar in *Pdgfrα* ^*+/+*^, *Pdgfrα* ^*+/flox*^, and *Pdgfrα* ^*flox/flox*^ animals, as well as the proportion of HFs with YFP^+ve^ cells in the different structures of the dermal compartment of the HF (Fig. 1a–d, e). The mean number of YFP^+ve^ cells present in the CTS, DC, or DP did not significantly differ between genotypes (Fig. 1a–d, f).

**Fig. 2:**
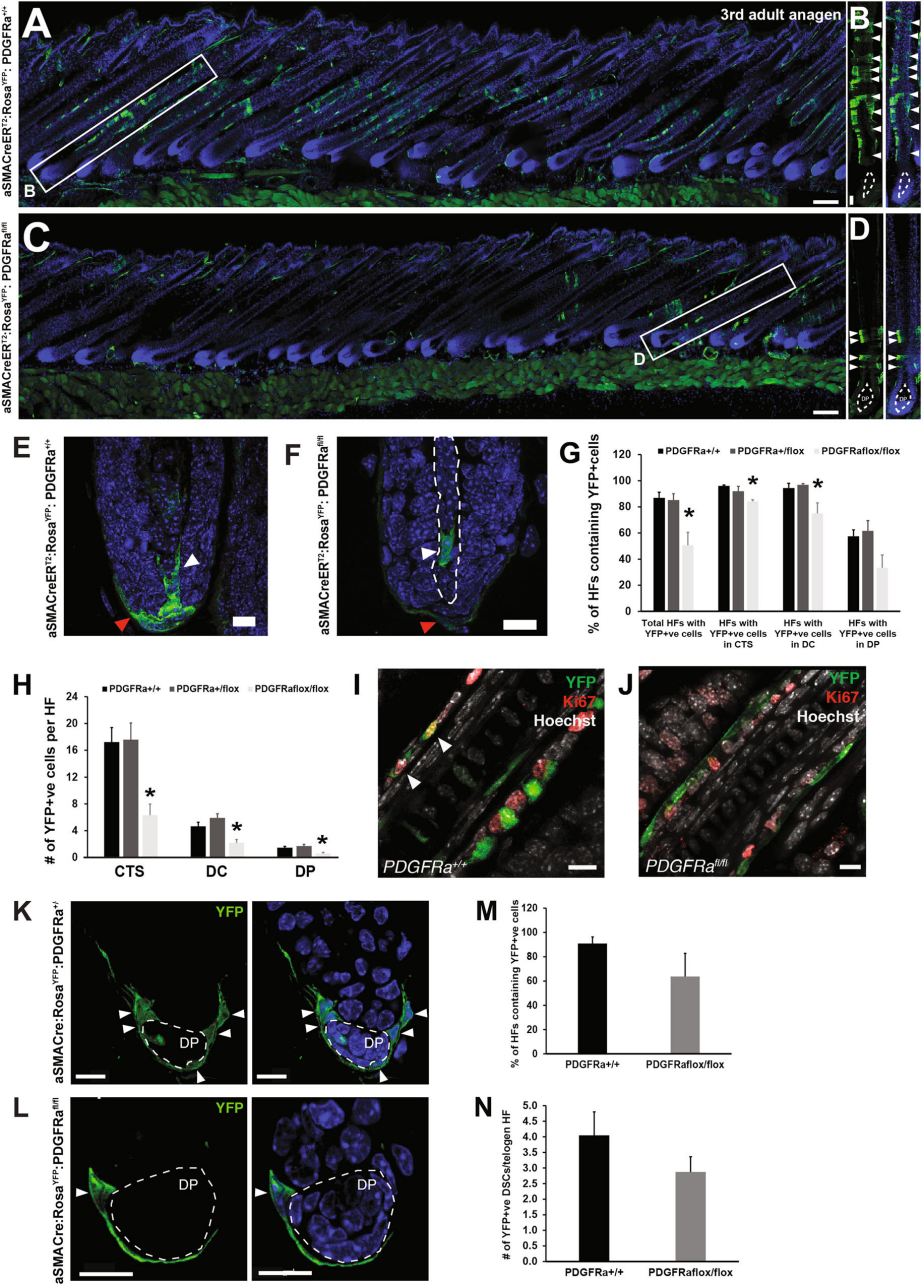
*Pdgfrα-*deficient hfDSCs exhibit progressive loss of progeny over consecutive hair cycles and a diminished stem cell pool. **a**,**b** Images of dorsal backskin from αSMACreER^T2^ *:*Rosa^YFP^:*Pdgfrα* ^*+/+*^ or **c**, **d** *Pdgfrα* ^*flox/flox*^ animals during third anagen (P60–62). **b** Magnified view of an individual HF from *Pdgfrα* ^*+/+*^ skin (*white box* in (**a)**) showing YFP^+ve^ cells (*green*) in CTS (*arrowheads*). **d** Magnification of a HF from *Pdgfrα* ^*flox/flox*^ skin (*white box* in (**c**)) showing that only a small number of YFP^+ve^ hfDSC progeny have integrated into CTS (*arrowheads*) vs. the control. **e** Detail of YFP^+ve^ cells in the DP, outlined by *dashed lines* (*white arrowheads*) and DC (*red arrowhead*) compartments of a HF from *Pdgfrα* ^*+/+*^ or **f** *Pdgfrα* ^*flox/flox*^ animals. **g** Percentage of total YFP^+ve^ HFs in *Pdgfrα* ^*+/+*^ vs. *Pdgfrα* ^*flox/flox*^ animals. The percentage of HFs containing YFP^+ve^ cells in each dermal compartment of the HF are also included. **h** Average number of YFP^+ve^ cells per HF present in the dermal compartments of the HF in *Pdgfrα* ^*+/+*^; *Pdgfrα* ^*+/flox*^, and *Pdgfrα* ^*flox/flox*^ animals. Data are mean ± SEM (*n* = 17 animals) and were analyzed using a Kruskal–Wallis, followed by a Mann–Whitney *U* test. *Asterisks* denote at least *p* ˂ 0.05. **i**, **j** Images depicting Ki67 immunostaining of anagen HFs from *Pdgfrα* ^*+/+*^ (**i**) or *Pdgfrα* ^*flox/flox*^ (**j**) mice. Note, there was an absence of YFP^+^Ki67^+^ proliferative cells in *Pdgfrα* ^*flox/flox*^ follicles. **k–n** Diminished retention of hfDSCs following HF regression in *Pdgfrα*-mutant mice. Images showing YFP^+ve^ hfDSCs retained in the DC (*white arrowheads*) of second telogen HFs, surrounding the DP (dashed line) from *Pdgfrα* ^*+/+*^ (**k**) or *Pdgfrα* ^*flox/flox*^ (**l**) animals. The backs of the animals were depilated at P21 (telogen) and tamoxifen was administered by gavage at P21, P23, and P25 (1 mg/day/animal). Skin was harvested when animals entered the telogen phase after depilation (second adult telogen, at P45-P49) to quantify the number of hfDSCs retained for each genotype after tamoxifen Cre-induced recombination. **m** Total percentage of HFs containing YFP^+ve^ cells retained during the second adult telogen. **n** Mean number of YFP^+ve^ hfDSCs per HF present in the DC in *Pdgfrα* ^*+/+*^ vs. *Pdgfrα* ^*flox/flox*^ animals during the resting telogen phase. For each animal, mean values were calculated after evaluating 50 YFP^+ve^ HFs per animal. Data are mean ± SEM (*n* = 4 animals). Mann–Whitney U test (*p* = n.s.). Scale bar represents 100 (**a**, **c**); 30 (**b**, **d**), 10 (**e**, **f**, **k**, **l**) and 5 µm (**i**, **j**). See also Fig. S1

Despite the fact that there was no difference in HF density between genotypes, examination of hfDSCs after third anagen (~P60) revealed a significant decline in the total number of HFs containing YFP^+ve^ cells in *Pdgfrα* ^*flox/flox*^ animals (50.5 ± 9.90%) in comparison to the *Pdgfrα* ^*+/+*^ (86.8 ± 4.37%) (*p* < 0.05) (Fig. 2a–f). Furthermore, we observed significant differences between genotypes in the percentage of HF that contained YFP^+ve^ cells in the each of the dermal compartments (Fig. 2g) and more specifically, a significant decline in the number of YFP-labeled cells within each mesenchymal compartment in *Pdgfrα* ^*flox/flox*^ mice (Fig. 2h). The average number of YFP^+ve^ cells in the CTS during third adult anagen was 17.2 ± 2.2 cells per HF in the PDGF^+/+^ animals vs. 6.3 ± 1.6 cells per HF in *Pdgfrα* ^*flox/flox*^ mice (*p* < 0.001). Similarly, the mean number of YFP^+ve^ cells per HF in the DC was 4.6 ± 0.6 in PDGF^+/+^ and 2.2 ± 0.5 in *Pdgfrα* ^*flox/flox*^ mice (*p* < 0.05). The mean number of YFP^+ve^ cells that were recruited into the DP was also statistically lower in *Pdgfrα* ^*flox/flox*^ mice (0.6 ± 0.18) than in PDGF^+/+^ animals (1.5 ± 0.19) (*p* < 0.05; Fig. 2h). No differences were observed between wild-type (*Pdgfrα* ^*+/+*^) and heterozygous (*Pdgfrα* ^*flox/+*^) animals for the floxed allele in any of the parameters evaluated (Fig. 2g–h), indicating that a single *Pdgfrα* allele was sufficient to maintain normal hfDSC function. We also performed immunostaining of mid-anagen follicles for the proliferative marker Ki67 and found that although a small percentage (ranging from 1 to 5%) of YFP^+ve^ CTS cells were dividing in *Pdgfrα* ^*+/+*^ follicles at this stage (Fig. 2i), there was a complete absence of YFP^+^Ki67^+ve^ cells in *Pdgfrα* ^*flox/flox*^ follicles (Fig. 2j)*.*

As PDGF deficiency resulted in a progressive decline in YFP^+ve^ hfDSC progeny over two consecutive cycles, we hypothesized that this may be due to a reduction in the number of hfDSCs that are retained in the resting telogen follicle. Thus, we analyzed telogen follicles preceding the third anagen phase. Excision of the *Pdgfrα* floxed allele caused a reduction in the number of resting hfDSCs retained within the individual second telogen follicles (P45–49) (Fig. 2k–l). In *Pdgfrα* ^*flox/flox*^ mice, ~64% of HFs retained YFP^+ve^ hfDSCs in the DC compared to 91% of HFs in *Pdgfrα* ^*+/+*^ littermates (Fig. 2m). As well, the mean number of hfDSCs retained within the DC was reduced by 30% in the *Pdgfrα* ^*flox/flox*^ animals respective to the wild-type controls (2.8 ± 0.5 vs. 4 ± 0.8 YFP^+ve^ cells, respectively; Fig. 2n). Suspecting that this might be due to effects on cell survival, we stained for cleaved caspase-3 in late anagen skin, expecting to find an increased frequency of dying YFP^+^ cells in *Pdgfrα* ^*flox/flox*^ skin. Positive staining was confirmed in catagen control skin (Supplementary Fig. 2A), but we failed to find any YFP^+^Caspase-3^+^ cells in late anagen follicles from either genotype (Supplementary Fig. 2B, C).

### PDGF enhances adult SKPs proliferation and self-renewal in vitro

**Fig. 3:**
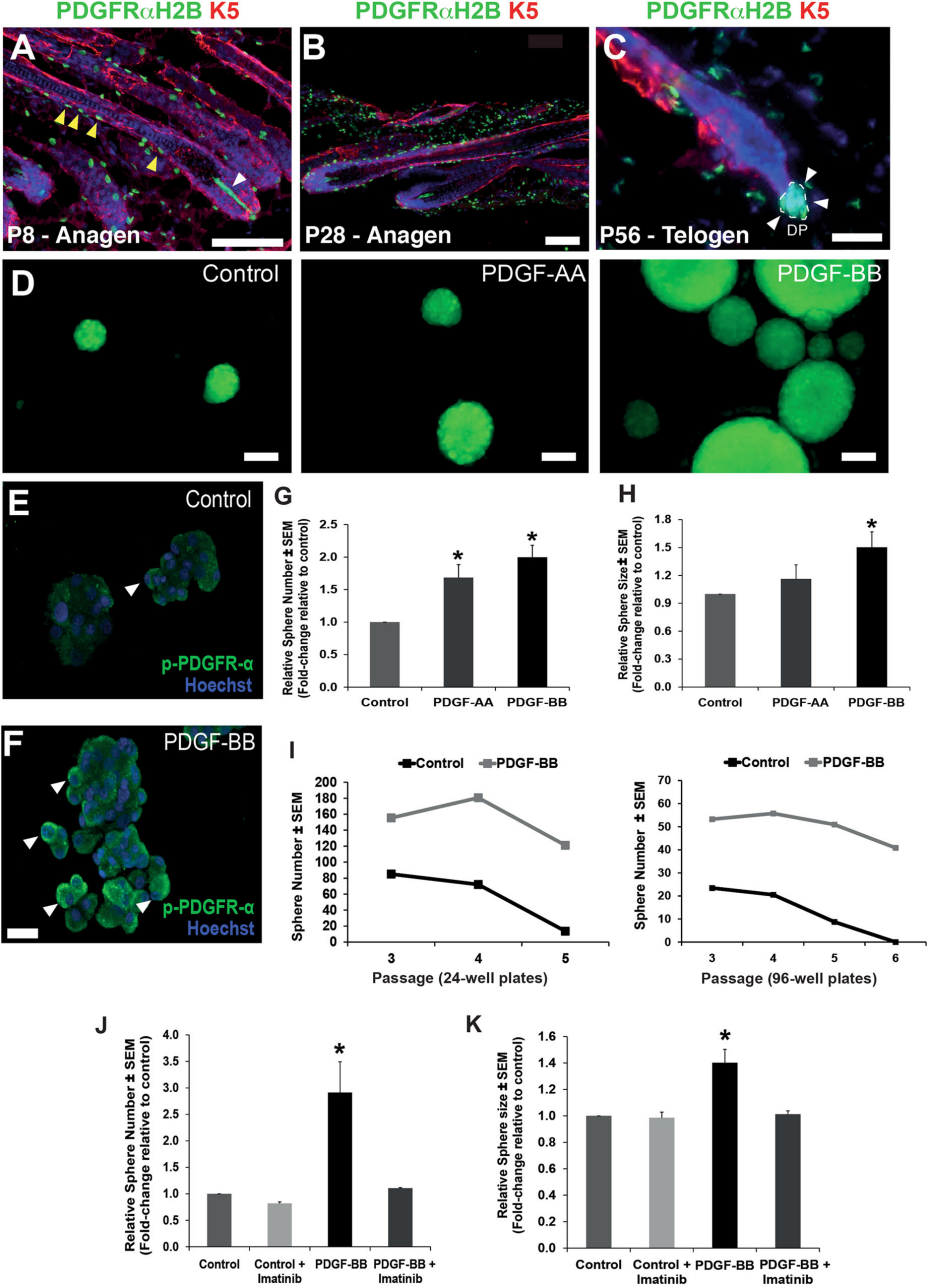
PDGF enhances adult rat SKPs proliferation and their self-renewal ability in vitro*.* **a** Immunofluorescence image of HFs at P8, first anagen (morphogenesis). *Yellow* and *white arrowheads* indicate PDGFRα-GFP^+ve^ cells (*green*) in the CTS and DP compartments, respectively. Keratin 5 (K5, *red*) labels the epithelial compartment of the HF. **b** Immunofluorescence image of HFs at P28, during the second anagen and; **c** immunofluorescence image of a HF at P56, during the second telogen. Note PDGFRα-GFP^+ve^ cells (*white arrowheads*) retained in the DP (*outlined*) during telogen. **d** Representative GFP^+ve^-labeled rat SKP spheres grown in the absence of PDGF (control) and in the presence of 50 ng/ml rat PDGF-AA or PDGF-BB. **e**, **f** Images of adult SKPs grown in the control conditions or with the addition of PDGF-BB and stained for phosphorylated PDGFRα (*green*). **g** Relative sphere number and **h** sphere size in the presence of PDGF-AA or PDGF-BB and controls. **i** Number of spherical colonies derived from rat SKPs cultured in the presence of the PDGF-B ligand (50 ng/ml) or control, over several passages (cultures were performed in 24-well plates, left chart; or in 96-well plates, right chart). **j** Relative sphere number and **k** sphere size in the presence of 50 ng/ml PDGF-BB and 1 µM Imatinib Mesylate. Kruskal–Wallis, followed by a Mann–Whitney *U* test (*asterisk* denotes at least *p* < 0.05). Scale bar represents 100 (**a**, **b**, **d**), 30 (**c**), or 25 µm (**e**, **f**). Nuclei are stained with Hoechst (blue). See Supplemental Information for immunofluorescence details

We previously demonstrated that hfDSCs are the cell of origin for SKPs which are self-renewing, Sox2+ colony forming cells that arise from dissociated dermal cells grown in vitro in the presence of bFGF.^3, 5^ We confirmed that *Pdgfrα* is present within the hfDSCs residing in the DC (Fig. 3a–c). We then asked how exposure to either PDGF ligand would impact growth of isolated SKPs. Based on this, we isolated SKPs from adult GFP rat skin and expanded them in standard SKPs growth medium, or in the presence of either PDGF-AA or PDGF-BB. By immunostaining for phosphorylated *Pdgfrα*, we confirmed that exposure to PDGF-BB was sufficient to activate *Pdgfrα* in cultured adult SKPs (Fig. 3e, f). The number of colonies was increased nearly 2-fold in the presence of PDGF-BB and 1.7-fold in the presence of PDGF-AA, when compared to the control conditions (Fig. 3d, g) (437 ± 31.1, 363.7 ± 10.9 vs. 227.8 ± 33.9 spheres, respectively; *p* < 0.05; *n* = 4 independent experiments). As an estimate of proliferative capacity and number of stem/progenitor cells in each colony, the size of the spherical colonies in each condition was also measured. Addition of PDGF-BB enhanced the mean colony size relative to controls by ~1.5-fold (84.6 vs. 58.2 µm, respectively; *p* < 0.05, *n* = 3 independent experiments) (Fig. 3h).

To determine the effect of PDGF on hfDSC self-renewal and therefore, their potential to maintain the stem cell pool, we performed serial passaging experiments on adult rat SKPs in the presence or absence of PDGF-BB (which showed the greatest effect on proliferation). SKPs grown under control conditions steadily declined in their ability to generate colonies over successive passages (Fig. 3i). However, addition of PDGF-BB maintained their ability to form spherical colonies with minimal decline over six passages (>60 days; Fig. 3g). Furthermore, SKPs cultured in the presence of PDGF-BB, were able to generate ≥2-fold more colonies than control at all-time points evaluated (Fig. 3i). Addition of the tyrosine kinase inhibitor Imatinib Mesylate to the culture medium completely abolished the effect of PDGF-BB on SKPs proliferation (Fig. 3j, k).

### PDGF-BB enhances proliferation of prospectively isolated adult hfDSCs

**Fig. 4:**
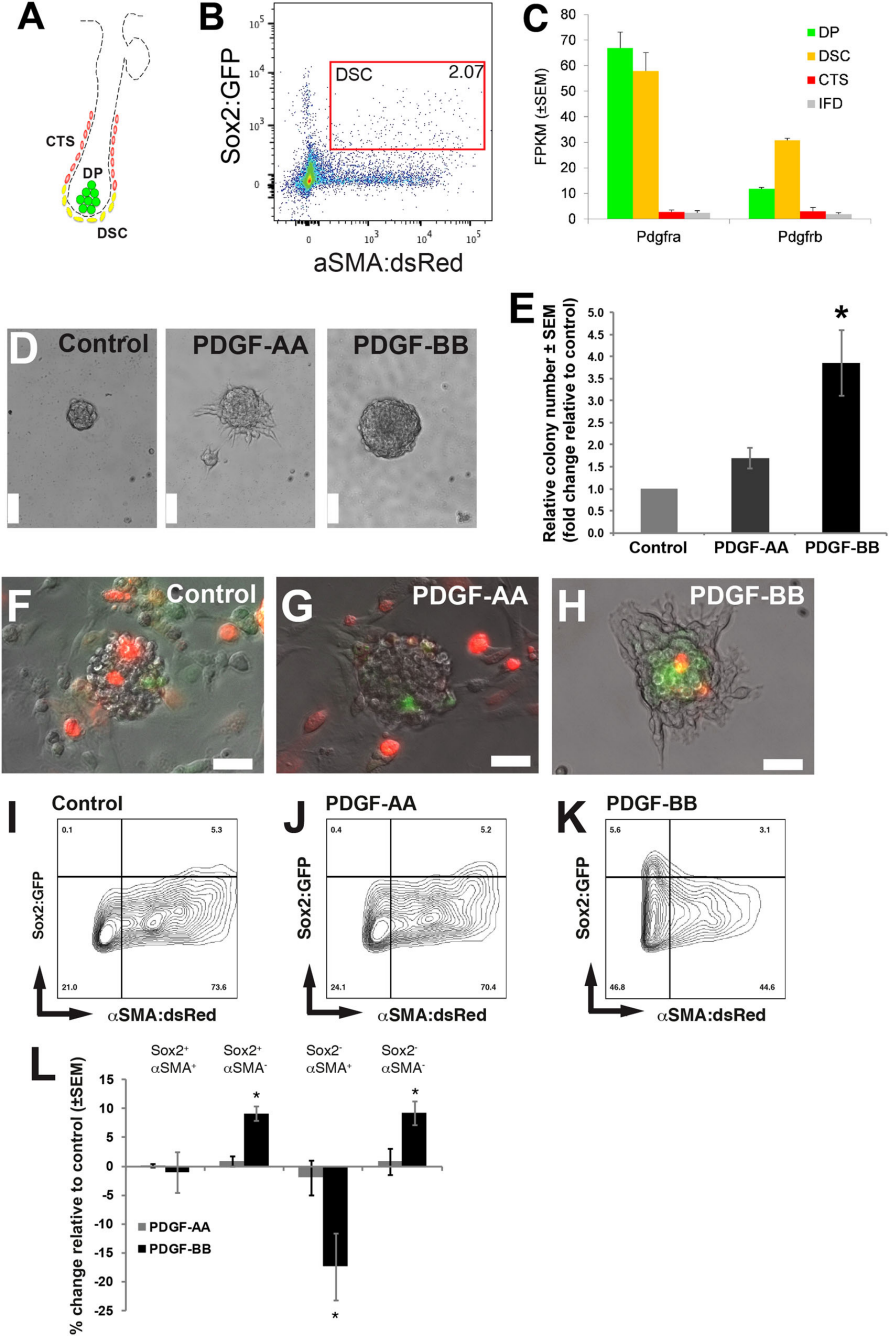
PDGF enhances expansion of prospectively isolated hfDSCs and promotes sustained expression of Sox2. **a** Schematic illustration showing Sox2^GFP^:αSMA^dsRed^ expression within the adult anagen HF. Dermal lineage compartments can be identified by their unique expression: hfDSCs (*yellow*), CTS (*red*), DP (*green*). **b** hfDSCs (Sox2GFP^+ve^ aSMAdsRed^+ve^ ; indicated by *red gate*) were prospectively isolated from P27 backskin by FACS. **c** Representative images of isolated hfDSCs grown in absence or presence of PDGF-AA or PDGF-BB. **d** Quantification of hfDSC colony number following exposure to PDGF-AA or PDGF-BB relative to control. **e–g** hfDSC-derived colonies were examined after 5 days for Sox2GFP and αSMAdsRed expression. **h-l** The frequency of expression within each population was quantified by flow cytometry. **I**. Change in Sox2GFP and αSMAdsRed expression relative to control after culture in the presence of PDGF ligands. Note the elevated frequency of Sox2GFP^+ve^ cells following exposure to PDGF-BB. Experimental data represent *n* = 3 independent experiments from three different groups of mice. Data are mean ± SEM and were analyzed with Kruskal–Wallis test. *Asterisk* denotes *p* < 0.05. Scale bars represent 50 µm.

We then asked whether PDGF treatment: (1) enhances proliferation of prospectively isolated hfDSCs, and (2) changes the fate choice of hfDSC progeny in vitro. Taking advantage of a Sox2GFP:αSMAdsRed double reporter mouse strain, we used FACS to identify and isolate primary adult hfDSCs (Fig. 4a, b) residing in the DC compartment, characterized as Sox2GFP^+ve^αSMAdsRed^+ve^ cells.^3^ First, we examined RNASeq expression data for *Pdgfrα* and *Pdgfrβ* in cells isolated from hfDSCs within the DC, DP, and CTS vs. interfollicular dermal fibroblasts (IFD). As expected, each of the dermal populations expressed both *Pdgf* receptors, however there was marked increase in transcripts for both *Pdgfrα* and *Pdgfrβ* in hfDSCs (and to a lesser extent DP), thus highlighting their potential importance in regulating hfDSC function (Supplementary Fig. 3; *n* = 3 biological replicates per cell type). To test this directly, isolated Sox2GFP^+ve^ αSMAdsRed^+ve^ (double positive) cells were then cultured in control conditions or with the addition of PDGF-AA or PDGF-BB, and assessed for effects on self- renewal /proliferation using a colony formation assay. After 5 days of growth, we found a significant increase in the average size of colonies (similar to that observed in rat SKPs; Fig. 4d), and a concomitant increase in the number of clonal colonies that were formed in the presence of PDGF-BB (Fig. 4e). We then asked whether PDGF treatment impacted hfDSC fate determination. To do this, we took advantage of our fluorescent reporter system, and used flow cytometry to assess the frequency of each possible phenotype within the hfDSC lineage (yellow, hfDSC; dsRed^+ve^, CTS; GFP^+ve^ DP) following 5 days exposure to PDGF-AA or PDGF-BB vs. control conditions (Fig. 4f–h). Interestingly, PDGF-BB showed a marked expansion of the Sox2GFP^+ve^αSMAdsRed^−ve^ population, not observed in either control or PDGF-AA conditions (Fig. 4i–l), suggesting that PDGF-BB may be maintaining an undifferentiated state or biasing the population to adopt a DP fate as opposed to CTS fate.

### Exposure to PDGF-BB maintains HF inductive capacity and bipotency of adult SKPs

**Fig. 5:**
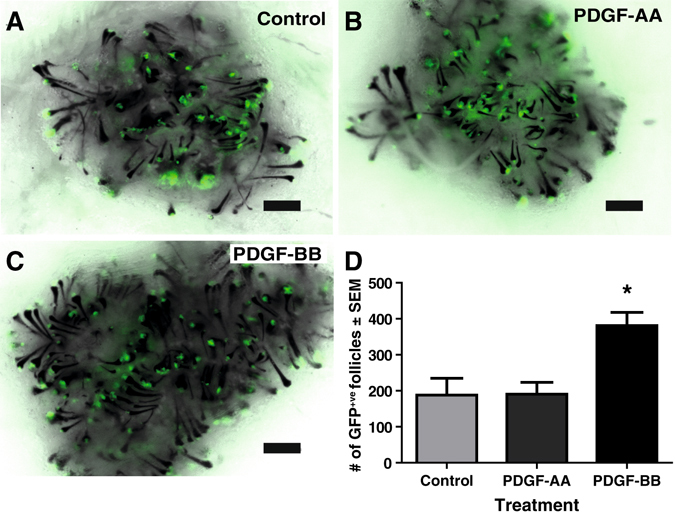
Exposure to PDGF-BB promotes enhanced inductive capacity of adult rat SKPs. Primary adult GFP-expressing rat SKPs were grown in the absence (control) or the presence of 50 ng/ml rat PDGF-AA or PDGF-BB. After one passage, SKPs colonies were dissociated to single cells and combined with epidermal aggregates (500,000 rat SKPs combined with 10,000 P0 mouse epidermal aggregates) and injected subcutaneously. Hair follicles were harvested and quantified 2 weeks post transplantation. Images show representative grafts from **a** control, **b** PDGF-AA, and **c** PDGF-BB. **d** Quantification of GFP^+ve^ HFs within each graft. Data represent three independent replicates, with a total of 4–6 grafts per group. Data were analyzed by one-way ANOVA. *Asterisk* denotes *p* ˂ 0.05. Scale bars represent 500 µm

Based on the increased frequency of Sox2^+ve^ hfDSC progeny when grown in PDGF-BB, we employed an ex vivo HF formation assay to specifically assess the impact of PDGF on the ability of SKPs to reconstitute the HF mesenchyme and to induce *de novo* HF formation. As we have done previously,^5^ we used GFP-expressing adult rat SKPs because of their superior in vitro growth and sustained inductive capacity relative to cultured mouse hfDSCs. Adult SKPs were grown in the presence or absence of PDGF and then combined with newborn mouse epithelial aggregates and transplanted subcutaneously in immune-deficient mice. Quantification of GFP^+ve^ follicles in each graft revealed that expansion of SKPs with PDGF-AA in the culture medium did not alter the subsequent ability of SKPs to induce HF formation when compared to controls (Fig. 5a, b, d). Intriguingly though, prior exposure to PDGF-BB significantly enhanced their ability to generate HFs (Fig. 5c, d) in comparison to the control (380.8 ± 37 vs. 188 ± 46.7; one way-ANOVA, *p* < 0.05). In all conditions, GFP-expressing SKPs had populated both the DP and CTS of newly formed HFs by 14 days, irrespective of treatment type (Fig. 5a–c).

### Exposure to PDGF enhances proliferation of adult human SKPs in vitro

**Fig. 6:**
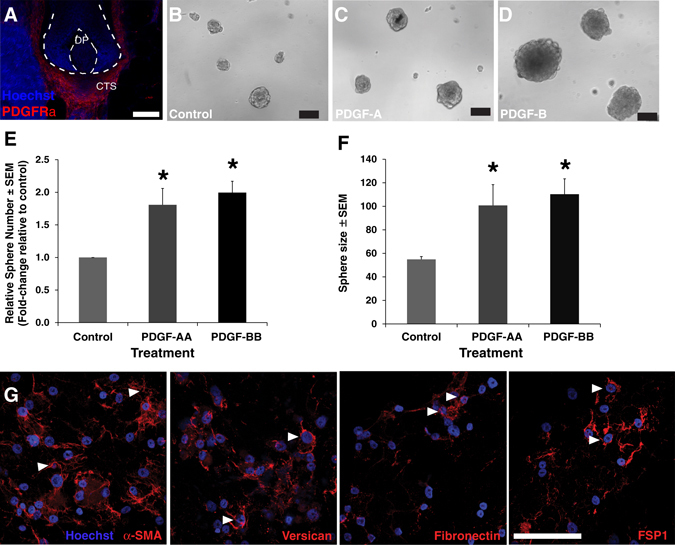
PDGF enhances adult human SKPs proliferation. **a** Image of an adult human scalp HF immunostained for PDGFRα. Note the absence of reactivity of the DP, but robust expression in the CTS compartment. Both the DP and the HF are outlined by dashed lines. **b** Human SKPs cultured in the absence of PDGF ligand or treated with 50 ng/ml **c** hPDGF-AA or **d** hPDGF-BB. **e** Fold-change in spherical colony number relative to control, formed after culturing human SKPs in the absence or presence of PDGF-AA or PDGF-BB. **f** Colony size (diameter) of the spheres formed in the presence of PDGF-AA or PDGF-BB vs. control. Data were collected from a total of four adult (>27 years of age) human samples. Data are mean ± SEM. *Asterisks* denote *p* < 0.05; Kruskal–Wallis, followed by a Mann–Whitney *U* test. See also Supplemental Information for immunofluorescence details. Scale bars represent 100 µm

Although PDGFRα is robustly expressed in all mesenchymal compartments of the mouse HF (Fig. 3a–c), in humans, PDGFRα expression is largely limited to the CTS in anagen scalp follicles, and absent in the DP (Fig. 6a). As PDGFRα is also expressed in the primary hfDSC niche, the DC, we asked whether PDGF had a similar effect on proliferation and self-renewal of adult human SKPs (Fig. 6b–f). Exposure to either human PDGF-AA or PDGF-BB increased the number of spherical colonies formed relative to control conditions (*p* < 0.05) (Fig. 6b–d, e). Similarly, the average size of the colonies was significantly larger in the presence of either PDGF ligand (*p* < 0.05; Fig. 6b–d, f), suggesting that PDGF signaling modulates the function of human hfDSCs as well. Moreover, the phenotype of human SKPs exposed to PDGF was not altered based on the expression of specific markers, such as versican, αSMA, fibronectin, and FSP1 (Fig. 6g).

## Discussion

Identifying the signaling pathways that govern self-renewal and fate choice of hfDSCs will be critical towards understanding their role in pathogenesis of skin disorders such as alopecia and/or development of cellular therapies to regenerate dermal tissue following severe skin injury. In the present study, we show that PDGF signaling is an important contributor to maintenance and proliferation of adult hfDSCs within the HF niche in vivo and can be used to robustly expand isolated hfDSCs in vitro, while maintaining an enhanced inductive capacity.

By genetically ablating *Pdgfrα* from hfDSCs in adult skin, we observed a marked depletion of hfDSCs and their progeny (in CTS and DP) over successive HF regenerative cycles, suggesting that PDGF signaling may stimulate proliferation of hfDSCs and CTS progenitor progeny. As the bulk of hfDSC progeny are required to regenerate the CTS, it is not surprising that there was such a pronounced reduction in YFP^+ve^ CTS cells. Indeed, our in vitro data for both prospectively isolated hfDSCs and SKPs support a role for PDGF signaling in hfDSC proliferation, as the number of colonies and size of colonies were elevated when grown in the presence of PDGF-BB (and to a lesser extent, PDGF-AA).

As well, the robust effect of PDGF-BB on serial passaging of adult SKPs suggests that it indeed promotes improved self-renewal of hfDSCs and maintenance of the stem cell pool. As HFs transition through the degenerative catagen stage and return to telogen, hfDSCs are retained around the periphery of the DP.^3^ Our finding that *Pdgfrα* ^flox/flox^ mice exhibited a 30% reduction in the number of YFP^+ve^ hfDSCs at second telogen compared to *Pdgfrα* ^+/+^ mice (prior to the reduction number of hfDSC progeny at third adult anagen) suggests that there is an initial decline in size of the stem cell pool, which subsequently results in fewer YFP^+ve^ progeny that repopulate the HF mesenchyme during regeneration. Recombination in these mice is incomplete and does not occur in all hfDSCs or their derivatives. This likely allows remaining *Pdgfrα* ^*flox/flox*^ cells (that have failed to undergo recombination as indicated by the lack of YFP expression) to outcompete *Pdgfrα*-deficient cells (YFP^+ve^ cells). Although there is a striking depletion of YFP^+ve^ cells, resulting from *Pdgfrα* deficiency, this compensatory response likely prevents a gross hair defect. Using in vivo clonal analysis of hfDSCs, we have described the functional heterogeneity that exists within the hfDSC pool,^3^ which has also been shown in the intestinal crypt.^20^ Such heterogeneity may be a consequence of competition for specialized niche factors, such as PDGF.

Maintenance of the stem cell pool within this system is likely heavily reliant on factors that support hfDSC survival, particularly as only a subset are retained following catagen degeneration. Thus, PDGF may also promote survival of hfDSCs in order to maintain the stem cell pool following each cycle and over successive cycles. Certainly previous work has demonstrated that PDGF-BB acts to promote survival of intestinal epithelial stem/progenitors by upregulating the serine/threonine kinase (AKT) activity.^21^ We failed to observe any apoptotic death within anagen HF mesenchyme (when DSCs are actively generating the CTS and contributing to the DP) in either *Pdgfrα* ^*+/+*^ or *Pdgfrα*-deficient follicles. Future studies may need to specifically examine the transition from catagen to telogen to determine whether PDGF is also protecting DSCs during HF regression. We observed a reduction in DSC number retained within the intermediate telogen, suggesting that this may be the case, in addition to their marked effect on self-renewal and proliferation. Furthermore, *Pdgfrα*-deficiency may also cause accelerated senescence of hfDSCs, as has been suggested by previous in vitro work showing that co-activation of PDGF and PTEN could prevent senescence of SKPs.^9^ This could also account for the limited cell division observed following *Pdgfrα*-deficiency and is something that should be addressed in future studies.

It is interesting that PDGF-BB had a positive effect on SKPs proliferation in vitro*.* Because the BB ligand activates both PDGFRα and PDGFRβ, the β-receptor might also exert a role in adult hfDSCs. Both α-receptors and β-receptors initiate common signaling pathways, although they each engage specific downstream effectors, having specific cellular effects.^22, 23^ In our hands, antibody detection of PDGFRβ within the HF mesenchyme has been unconvincing, however, our RNA sequencing data (Hagner and Biernaskie, unpublished data) shows that both *Pdgfrα* and *Pdgfrβ* are highly enriched in hfDSCs and DP relative to either CTS or IFD. Thus, it is possible that PDGFβ may also modify hfDSC behavior in vivo, but this remains to be determined.

Our observation that PDGF-BB treatment appeared to also bias fate choice of prospectively isolated hfDSCs and their progeny in vitro toward a Sox2^+ve^ phenotype is intriguing. Although not necessary for the inductive function of DP cells,^24^ Sox2^+ve^ aSMA^−ve^ expression is highly indicative of the endogenous hfDSC and DP phenotypes (in Awl, Auchene and guard HFs), suggesting that PDGF signaling may influence acquisition of the DP fate. Indeed, our transplantation and *de novo* HF formation assay confirmed that prior exposure to PDGF-BB greatly improved the inductive capacity of SKPs, leading to greater reconstitution efficiency. Together, these findings strongly suggest PDGF-BB either promotes acquisition of the DP fate or prevents differentiation of hfDSCs, which would allow sustained self-renewal and overall proliferative capacity.

Previous work analyzing PDGF-A and *Pdgfrα* null mutants revealed the importance of PDGF-A and *Pdgfrα* in epithelial–mesenchymal interactions^19, 25–29^ during development. The PDGF family is critical during embryonic development and conventional loss-of-function mutations for both PDGF-A and PDGF-B ligands^19, 30^ and *Pdgfα* and *β-*receptors^17, 31^ are nearly always lethal, or confounded by systemic effects. Previous studies reported a role for PDGF-A and *Pdgfrα* in the dermal compartment of the HFs during morphogenesis.^19, 32^ Mice carrying a null allele for PDGF-A displayed misshapen follicles with abnormal CTS, shrunken DP and dermal thinning relative to their wild-type counterparts.^19^ Additionally, administration of a *Pdgfrα* neutralizing antibody into newborn mice also perturbed HF formation.^32^ Contrary to this, recent work specifically evaluated the role of PDGF signaling in HF induction and morphogenesis, performing conditional ablation of both *Pdgfrα* and *Pdgfrβ* within the DP (using a TbxCre promoter) during late embryonic development.^18^ Surprisingly, conditional *Pdgfrα/β* deletion failed to alter HF induction or formation, but did result in dermal hypoplasia.^18^ This suggests that PDGF signaling is dispensable for DP inductive function during HF morphogenesis, but is important for maintaining dermal fibroblasts postnatally. Indeed, previous studies using PDGF-A and *Pdgfrα* null mice^19^ showed similar findings, however also noted that HFs failed to enter the second anagen growth (i.e., first adult anagen). Our data show that this may be due to the impact of PDGF deficiency on hfDSCs and their inability to generate sufficient numbers of either DP or CTS cells during HF regeneration. It should also be noted that our studies used depilation to synchronize HF regeneration. Thus, it may be that PDGF ligands act to promote rapid proliferation of DSCs during injury-induced hair growth. Future studies, may want to examine the role of PDGF signaling in DSC function during normal cycling. Nevertheless, our results show that DSCS are highly sensitive to PDGF signaling and that in the absence of *Pdgfrα* DSCs exhibit a diminished capacity to populate the mesenchymal pool within each HF. Conversely, exposing prospectively isolated DSCs (and both rodent and human SKPs) to PDGF results in a marked stimulation of proliferation and self-renewal strongly suggesting that endogenous PDGF signaling is an important regulator of DSC function within their HF niche.

In our study, both PDGF-AA and PDGF-BB had a positive effect on the proliferation of rat SKPs based on the size and the number of spheres formed in the presence of PDGF in relation to the control without PDGF, although this positive effect was more pronounced in the presence of PDGF-B ligand. This parallels previous work showing that PDGF increased proliferation of isolated DP cells in vitro.^33^ Conversely, other studies described that PDGF-AA stimulated the growth of cultured rat fibroblasts, but not rat vibrissa DP cells that was consistent with a higher expression of PDGFRα in fibroblasts than in DP cells.^34^ Members of the PDGF family have previously been identified in human skin samples. Both PDGF-A and PDGF-B ligands were detected in several compartments of fetal human skin as well as both PDGF receptors.^35^ The follicular sheath was immunoreactive with both anti-PDGFRα/β antibodies, whereas the DP cells showed no reaction.^35^ PDGF-A mRNA was detected in cultured human follicular keratinocytes and DP cells, whereas the expression of PDGF-B was only detected in follicular keratinocytes. Conversely, human follicular keratinocytes expressed both types of PDGF receptors, but DP cells only expressed PDGFRβ on the protein level.^36^ Our results confirm that PDGFRα is robustly expressed within the proximal CTS (the hfDSC niche), but not in the DP of human HFs, and that both PDGF-AA and PDGF-BB ligands stimulated growth of adult human SKPs in vitro. This is in agreement with a previous report showing that PDGF-AA promotes spherical colony formation of human adult SKPs.^9^ The observed mitogenic effect of PDGF-AA was potentiated in the presence of a PTEN inhibitor, and was proposed to act by alleviating stem cell senescence and promoting the self-renewal of human SKPs through the PI3K-Akt pathway.^9^ Regardless of the mechanism of expansion, PDGF also does not appear to alter the inherent SKP phenotype as demonstrated by sustained expression of previously characterized markers,^3, 5, 9^ which is an important consideration as a potential exogenous source of dermal progenitors for skin/connective tissue repair.

The source(s) of PDGF within the hfDSC niche remains elusive. Reticular dermal adipocytes,^37^ epidermal keratinocytes,^38^ and more recently senescent dermal fibroblasts have all been suggested as local sources of PDGF-AA within the adult skin.^12^ Future studies will need to identify the cellular source of PDGF as well as the complement of receptors within hfDSCs as alteration of PDGF signaling appears to have drastic effects on hfDSCs function and may contribute to pathological hair loss or dermal wound healing.

In summary, our study revealed that deficiency of *Pdgfrα* within hfDSCs causes a progressive decline in the hfDSC pool and subsequent diminished contribution to both DP and CTS. Exposure to PDGF (AA or BB)-enhanced proliferation and long-term expansion of isolated rodent hfDSCs and human SKPs, while maintaining their inherent inductive function and bipotency. Overall, this work suggests that PDGF may be an important additive to rapidly expand hfDSCs ex vivo for cell-based therapeutic applications, and that disruption of PDGF signaling within the adult hfDSC lineage may contribute to the pathogenesis of hair loss.

## Methods

All experiments including animals were performed in accordance to the established guidelines by the Canadian Council of Animal Care and were approved in advance by the Health Sciences Animal Care Committee (HSARC) from the University of Calgary.

### In vivo fate mapping of PDGFRa-deficient hfDSCs

As shown previously, αSMACreER^T2^:Rosa^YFP^ allows exclusive long-term labeling of hfDSCs and their progeny over multiple regenerative HF cycles.^3^ To examine the role of *PDGFRα*, we crossed the *Pdgfrα* ^*flox*^ strain with the αSMACreER^T2^ *:*Rosa^YFP^ to specifically label hfDSCs and their progeny while conditionally deleting PDGFRα within the HF mesenchymal lineage. Mice from each genotype were systematically matched by gender and body weight, and then subsequently designated to each group. In order to synchronize onset of anagen across all animals, backskin was depilated (by plucking) at P21 (telogen). Tamoxifen (4-OHT; Sigma Aldrich) was given by gavage at P21, P23, and P25 (1 mg/day/animal) to all animals (regardless of genotype) in order to control for potential non-specific effects of tamoxifen on HF growth. Dorsal skin was harvested at second anagen (P33-P35); second telogen (P45-P49), and third anagen (P60-62) for immunostaining (See Fig. S1 and Supplemental Material). Skin was fixed overnight in 2% paraformaldehyde, followed by a sucrose gradient (10, 20, and 30% sucrose in PBS) every 8–16 h before being embedded in optimum cutting temperature (OCT) compound, snap frozen in methylbutane and stored at −80 °C. Skin was sectioned and evaluated using a spectral confocal microscope (Leica TCS SP8, Leica Mycrosystems Inc.). The thickness of each section was 55 µm, and each slide was prepared so that individual sections were at least 275 µm apart from each other. The percentage of HFs that contained YFP^+ve^ cells was determined. For each animal, we calculated mean values after evaluating 50 HFs per animal and quantified the number of YFP^+ve^ cells present in each compartment of the HF: (i) the DP, (ii) the DC surrounding the follicle bulb (defined as the cells in the CTS below Auber’s line), and (iii) the CTS (defined as the cells in the sheath above Auber’s line up to the bulge/arrector pili muscle) per follicle. In skin samples collected in telogen, the number of YFP^+ve^ hfDSCs retained per HF in the DC were recorded.

### Adult rat SKPs isolation and in vitro expansion

To examine the role of PDGF on hfDSC function, we first used adult rat SKPs (which originate from endogenous hfDSCs)^5^ because they exhibit superior growth in vitro and inductive capacity in ex vivo HF formation assays relative to mouse SKPs.^5, 39^ Rat SKPs were isolated from the back skin of 30–33 day-old GFP-expressing Sprague Dawley rats (SLC, Japan) as described.^39^ Briefly, isolated dorsal skin, free of hair fibers and subcutaneous fat, was placed in 5 mg/ml dispase (StemCell Technologies) for 1 h at 37 °C followed by mechanical removal of the epidermis. The remaining dermal layer was minced into 1–2 mm pieces and placed in 1 mg/ml collagenase (type XI, Sigma Aldrich) for 1 h at 37 °C with agitation every 15 min. The supernatant containing isolated SKPs was removed, filtered through a 40 µm mesh and the cells were washed twice in Hank’s balanced salt solution (HBSS, Invitrogen) and pelleted by centrifugation (1200 rpm; 5 min). Primary cultures were seeded at an initial density of 50,000 cells/ml in 75 cm^2^ flasks and subsequent passages were grown at 10–20,000 cells/ml to promote clonal colony formation. SKPs proliferation medium consisted of DMEM and F12 (3:1,v/v; Invitrogen) containing 40 ng/ml bFGF (BD Biosciences), 20 ng/ml epidermal growth factor (BD Biosciences), 2% B27 (Invitrogen), and 1% penicillin-streptomycin (Invitrogen).

### *In vitro* assessment of PDGF signaling on rat SKPs proliferation and self-renewal

To evaluate the effect of PDGF on SKPs proliferation and their ability to self-renew, rat SKPs were used at passage 2. For passaging, rat SKPs colonies were incubated in 1 mg/ml collagenase (type XI, Sigma Aldrich) at 37 °C for 10 min, to dissociate the spheres into single cells. Cells were plated in 24-well plates at 10,000–20,000 cells/ml and grown in culture medium alone or with the addition of 50 ng/ml rat PDGF-A or PDGF-B ligands (R&D Systems Inc., Minneapolis, USA) for the duration of the experiment. Cell feeding was performed every three days. Depending on their growth rate, 9–20 days later, the number of spheres was quantified and each well was imaged using an Axioplan inverted microscope (Zeiss). The size (diameter) of spherical colonies was quantified using ImageJ software (http://imagej.nih.gov/ij/). For all the experiments, an average value was obtained from several wells per treatment for each replicate (at least three independent experiments). The number and size of spheres were normalized to each respective control to account for differences in cell density and cell line variability and expressed as the fold-change relative to control. In order to assess self-renewal, a serial colony formation assay was performed. Cells were grown at 20,000 cells/ml in 24-well or 96-well plates (one independent experiment for each) in the presence or absence of 50 ng/ml PDGF-BB. The number of colonies formed per treatment was quantified 12–20 days after each passage up to passage 5–6. The number of days between passages was increased over culture due to the decline in speed growth over passages. To confirm the signaling underlying the effect of PDGF on rat SKPs proliferation, a tyrosine kinase inhibitor, Imatinib Mesylate (sc-202180, Santa Cruz Biotechnology, Dallas, Texas, USA), was used. Rat SKPs were treated with 1 µM Imatinib Mesylate, with or without PDGF-BB. Imatinib was added and allowed to incubate for 45–60 mins prior to the addition of the proper feeding regimen. Spherical colony size and the number of colonies were quantified 7–8 days later to assess differences in cell proliferation as indicated above. For each replicate (*n* = 3–5), a mean value was obtained from at least three wells per treatment. To assess activation of PDGFRα, rat SKPs grown in presence or absence of PDGF-BB ligand for 8 days were immunostained for phospho-PDGFRα (1:200; Santa Cruz Biotechnology, CA, USA). Immunofluorescence staining was imaged using an Axioplan inverted microscope (Zeiss) where exposure time was kept consistent between both conditions.

### Prospective isolation of hfDSCs

Cells from the DC compartment were obtained from the back skin of Sox2EGFP:αSMAdsRed (crossed in our lab)^40, 41^ mice at the onset of the second adult anagen (P26–28). A sorting strategy for isolating cells from the several mesenchymal compartments of the HF has been described.^3^ Briefly, DP cells were characterized as Sox2:GFP^+ve^/αSMAdsRed^−ve^; cells from the CTS were characterized as Sox2:GFP^−ve^/αSMAdsRed^+ve^; and cells from the DC were considered as double positive (Sox2:GFP^+ve^ and αSMAdsRed^+ve^). A BD FACSAria^TM^ III (Becton Dickinson, Canada) was used. Viable cells were identified by Fixable Viability Dye eFluor 780 (eBioscience, 65-0865-14). Double positive-sorted cells were plated at 10,000 cells/ml and cultured in vitro as indicated for rat SKPs. Cells were grown in control conditions or in the presence of 50 ng/ml PDGF-AA or PDGF-BB. Sphere numbers were quantified on day 5 after sorting. To evaluate the effect of PDGF ligands on DC cells fate decisions, colonies were dissociated to single cells and analyzed by flow cytometry to analyze the frequency of each phenotype within the hfDSC lineage. Each independent replicate (*n* = 3) was performed with pooled cells harvested from 2–3 transgenic mice.

### *Ex vivo* HF formation assay

We used the “patch” HF formation assay^5, 39^ (see also Supplemental Material) to assess the impact of PDGF on the ability of isolated hfDSCs to stimulate new HF formation. After 14 days, grafts were harvested, and the number of HFs containing GFP^+ve^ DPs in each graft were quantified (*n* = 3, with 4–6 grafts per treatment).

### Isolation and culture of human SKPs

Adult human scalp skin was obtained from deceased organ donors via the Southern Alberta Tissue Recovery and Transplant Program. Informed consent from next of kin was provided and all procedures received prior approval from the University of Calgary Conjoint Human Research Ethics Board. Skin was washed with HBSS; the epidermis was placed side up and wiped with tissue moistened with 70% ethanol. Tissue was placed into fresh HBSS and cut with a scalpel into 2–4 mm strips and subsequently digested in dispase (5 U/ml; StemCell Technologies) at 37 °C for 4 h. The epidermis was separated from the dermis with forceps after the incubation was completed. Strips of dermis were then washed with HBSS to remove residual red blood cells. Dermis was minced with a scalpel and subsequently incubated in collagenase IV (1 mg/ml; Worthington) with F12 to a final volume of 40 ml. DNase (50 µg/ml) was added to prevent cell adhesion. Tissue was incubated for 4–5 h at 37 °C with intermittent trituration and cell harvesting occurring every hour during the incubation period. Viable cells were counted using Trypan blue exclusion and plated in 24-well plates at 20,000 cells/ml in SKPs proliferation medium as above for rat SKPs with or without PDGF-AA or PDGF-BB (50 ng/ml).

### Statistical Analysis

All data are represented as mean ± SEM. Data were analyzed with one-way ANOVA or Kruskal–Wallis tests for data sets that were not normally distributed. Statistical analyses were done using GraphPad Prism or Statistica (Statsoft, Inc. version 6; www.statsoft.com), and *p* < 0.05 was considered significant. Rat SKP proliferation experiments were done with at least three independent biological replicates with several experimental replicates per treatment. SKP self-renewal experiments were done in 24-well plates and 96-well plates (once each, thus, no statistical analysis was performed) with multiple wells per treatment. Prospective isolation of mouse hfDSCs and in vitro analysis was performed in triplicate using three different litters of transgenic mice. Ex vivo patch experiments were done in three independent replicate experiments with 4–6 grafts per treatment in each experiment. Quantification of YFP cell fate included wild-type (*n* = 3), heterozygous (*n* = 4) or *Pdgfra* ^*flox/flox*^ (*n* = 3) mice during the second adult anagen (Fig. 1); wild-type (*n* = 2) and *Pdgfra* ^*flox/flox*^ (*n* = 2) mice during the second adult telogen phase (Fig. 2), or wild-type (*n* = 7), heterozygous (*n* = 5) and *Pdgfra* ^*flox/flox*^ (*n* = 5) mice during the third adult anagen (Fig. 2). All quantifications were performed by an observer blinded to experimental conditions.

## Electronic supplementary material

Supplementary Information

Figure S1

Figure S2

Figure S3
