# Supplementary material for: Platelet-derived growth factor signaling modulates adult hair follicle dermal stem cell maintenance and self-renewal
Source: NPJ Regen Med. 2017 Apr 14;2:11. doi: 10.1038/s41536-017-0013-4 (PMC5665619; doi:10.1038/s41536-017-0013-4)
Supplement: Supplementary file 1 — Supplementary Information [file 41536_2017_13_MOESM1_ESM.pdf]

Supplemental information

1. Supplemental figures and legends

Figure S1. Experimental timeline, genotyping and confirmation of recombination in *αSMACreER^{T2}.Rosa^{YFP}.Pdgfrα^{flox}* animals

(**A**) Schematic of experimental timeline to evaluate the role of *Pdgfrα* in the dermal compartment of the hair follicle .The backs of the animals were depilated at P21 (telogen) and tamoxifen was administered by gavage at P21, P23 and P25 (1mg/day/animal). The subsequent anagen phase was documented and compared between tamoxifen-treated wild type (n = 3), heterozygous (n = 4) or *Pdgfrα$^{flox/flox}$* (n = 3), carrying Cre, animals. For these, skin was harvested during mid-anagen (P33-P35). In another set of animals, the next two consecutive-induced hair cycles were evaluated and compared between tamoxifen-treated wild type (n = 7), heterozygous (n = 5) or *Pdgfrα$^{flox/flox}$* (n = 5), carrying Cre, animals. A second depilation was performed at P50 (telogen) and dorsal skin was harvested at P60-62 (mid-anagen) for immunostaining. Based on the results observed from the mentioned collection time-points, we also collected skin from animals that entered 2nd adult telogen (harvested around P45-P49) from tamoxifen-treated wild type (n = 2) or *Pdgfrα$^{flox/flox}$* (n = 2), Cre^{+ve} animals. (**B**) Example of genotyping before tamoxifen administration using multiplex PCR with primer 1, primer 2 and primer 3 (see map). Lanes 1 and 4 depict wild-type genotype; lanes 2-3 and 5-6 represent heterozygous animals (*Pdgfrα$^{+/flox}$*) and lanes 7-10 represent homozygous animals for the floxed allele (*Pdgfrα$^{flox/flox}$*). Lane 11 is empty and lane 12 corresponds to the non-template control. Ladder is a 100 bp DNA ladder. (**C**) PCR example using primers 1 and 4 for the detection of the excision after tamoxifen admistration and Cre-mediated recombination of the floxed allele. Lanes 1-2: *Pdgfrα$^{flox/flox}$* and Cre negative controls; lane 3: *Pdgfrα$^{flox/flox}$* and Cre^{+} vehicle-administered control. Lanes 4-5: wild-type and

Cre+ tamoxifen-administered controls. Lanes 6-9 represent *Pdgfrα*$^{flox/flox}$ and Cre^{+ve} animals with successful recombination after tamoxifen administration. Note the presence of an approximately 600 bp band only in lanes 6-9, as expected, and indicative of excision of the floxed allele. Lane 10 is the non-template control. Ladder is a 100 bp DNA ladder. (**D**) Partial maps of the *Pdgfrα*$^{+/+}$ (wild-type), targeted floxed and the resulting lox allele after Cre-mediated inactivation of *Pdgfrα*. Black boxes represent exons. Red triangles represent *loxP* sites and the floxed allele contains the Neo cassette (gray box). Blue arrows represent the location of the PCR primers used to discriminate between wild-type and floxed alleles, as well as to evaluate Cre recombination and excision of the floxed allele after tamoxifen administration. Map adapted from Tallquist and Soriano [1]. (**E**) Sequence obtained (5'→ 3') after gel PCR-product DNA purification to confirm excision of the targeted floxed allele. A fragment of approximately 580 bp was obtained after sequencing, containing a fragment upstream of the exon 1 (primer 4 sequence highlighted in grey), followed by part of the BamHI site (highlighted in blue). After the BamHI site, the sequence contains part of the Soriano's targeting vector (red letters; NCBI GQ424832.1), which contained the loxP site (highlighted in light grey). The sequence continues with part of the SmaI site sequence (yellow), followed by a fragment downstream of the exon 4, up to primer 1 (highlighted in grey), which confirms successful recombination and deletion of exons 1 to 4 from the *Pdgfrα*-floxed allele.

Figure S2. Loss of *Pdgfrα* does not increase cell death of DSCs and their progeny. (**A**) Immunofluorescence image of an adult catagen hair follicle stained for Cleaved Caspase 3 (red; used as a positive control) is shown. The hair follicle is outlined by dashed lines and white arrowheads indicate Caspase 3^{+ve} cells. (**B, C**) Hair follicles from 2nd adult late anagen (P33-P35) were examined for co-expression of recombined YFP^{+ve} cells (green) for co-expression of Cleaved-Caspase 3 (red); representative immunofluorescence images of follicles from (**B**) *Pdgfrα*$^{+/+}$ and (**C**) *Pdgfrα*$^{flox/flox}$ animals are shown. Scale bar represents 50 µm (A-C).

Figure S3. Elevated expression of *Pdgfrα* and *Pdgfrβ* expression in prospectively isolated hfDSCs. Cells residing within the dermal cup (hfDSCs), DP, dermal sheath and interfollicular dermis were prospectively isolated from $Sox2^{GFP}$:$aSMA^{dsRed}$ mice and expression of (**A**) *Pdgfrα* and (**B**) *Pdgfrβ* was performed using real-time quantitative PCR. N=3 biological replicate samples for each group.

2. Supplemental experimental procedures

Mice strains and genotyping

$αSMACreER^{T2}$:$Rosa^{YFP}$:$Pdgfra^{flox}$ mice

Pdgfrα floxed animals were obtained from the Jackson laboratory repository (B6.Cg-$Pdgfrα^{tm8Sor}$/EiJ; 006492) and crossed with *$αsmaCreER^{T2}$:$Rosa^{YFP}$* mice. Since the connective tissue cells of the hair follicle express *Pdgfrα* and they are also alpha-smooth muscle positive, this breeding strategy allows us to selectively evaluate the role of *Pdgfrα* in the dermal sheath compartment. α-$SMACreER^{T2}$ mice [2] allow efficient, temporally-controlled mutation in almost all alpha-smooth muscle actin expressing cells [2] and were therefore bred with a RosaYFP reporter strain (007903 B6.Cg-Gt(ROSA)$26Sor^{tm3(CAG-EYFP)Hze}$/JJackson lab). This α-$smaCreER^{T2}$:$Rosa^{YFP}$ strain has been successfully used for *in vivo* fate mapping of hfDSCs. To genotype for Cre expression, the following oligonucleotides were used: TK139 (5'-ATT TGC CTG CAT TAC CGG TC-3') and TK141 (5'-ATC AAC GTT TTC TTT CGG A-3') as well as myogenin as an internal control: ADV28 (5'-TTA CGT CCA TCG TGG ACA GC-3') and ADV30 (5'-TGG GCT GGG TGT TAG CCT TA-3'). These primers were run under the following conditions: 94°C, 5 min; 94°C, 30 secs/ 55°C, 30 secs/ 72°C, 30 secs for 30 cycles; 72°C, 7 min. The obtained products are 350

bp for Cre and 230 bp for the internal control. For evaluating the expression of the YFP reporter, the following primers were used: wild type forward (5'-AAG GGA GCT GCA GTG GAG TA-3') and reverse (5'-CCG AAA ATC TGT GGG AAG TC-3'); mutant forward (5'-ACA TGG TCC TGC TGG AGT TC-3') and reverse (5'-GGC ATT AAA GCA GCG TAT CC-3') and run for 95°C, 2 min; 95°C, 30 secs/ 60°C, 30 secs/ 72°C, 40 secs for 35 cycles; 72°C, 8 min. The obtained products were: 212 bp for the homozygous mutants; 297 bp for the wild type and the two products for heterozygous mutant animals.

The *Pdgfrα*^{flox} allele was previously described [1,3]. Briefly, a *loxP* site was inserted into the *Bam*HI site upstream of exon 1 and a *PGK-neo loxP* cassette was inserted downstream of exon 4 at the *Sma*Isite. This construct carries a deletion of ~6.5kb that corresponds to the signal peptide as well as the first and second Ig domains of the gene. To identify the genotypes, PCR was performed on tail biopsies using the following primers: (P1) 5'-CCC TTG TGG TCA TGC CAA AC-3'; (P2) 5'-GCT TTT GCC TCC ATT ACA CTG G-3'; (P3) 5'-ACG AAG TTA TTA GGT CCC TCG AC-3' (Soriano, 1997). The PCR conditions were: 94°C, 2 min; 94°C, 30 secs/ 60°C, 30 secs/ 72°C, 40 secs for 30 cycles; 72°C 8 min. This results in the amplification of a 242 bp product that corresponds to the wild-type (*Pdgfrα*$^{+/+}$) and a 451 bp product that corresponds to the floxed allele (*Pdgfrα*$^{flox/flox}$). Heterozygous animals (*Pdgfrα*$^{+/\text{flox}}$) will display two bands corresponding to the amplification of both products. To determine if successful recombination occurred after tamoxifen administration, ear notches were collected 4-6 days after the last tamoxifen dose (see below). The primer P1 (downstream exon 4) was used together with (P4; upstream exon 1): 5'-CAC AAG AAC ACA GGC TGG AAG 3'-3' and were run using the PCR protocol: 94°C, 4 min; 94°C, 30 secs/ 65.5°C, 30 secs/ 72°C, 40 secs for 35 cycles; 72°C 8 min (see also supplemental figure S1, with the partial maps of the wild-type, floxed and lox allele). A product size of about 600 bp was obtained. The DNA was purified using a gel PCR-product DNA purification kit (QIAquick gel extraction kit #28704, Qiagen Sciences, Maryland, USA)

following the manufacture's instructions and the DNA was sequenced (Applied Byosystems 3730xl 96 capillary DNA analyzer; University of Calgary Core DNA Services) to confirm excision of the targeted floxed allele.

4-6 days following tamoxifen application, PCR genotyping and subsequent electrophoresis revealed an approximately 600 bp band in Cre^{+ve} animals containing the floxed allele (*Pdgfrα*$^{flox/flox}$), which was indicative of successful excision. The presence of this ~600bp amplicon was not observed in any of the samples from *Pdgfrα*$^{+/+}$ (wild type), carrying Cre or Cre negative tamoxifen-administered controls; *Pdgfrα*$^{flox/flox}$ *or* *Pdgfrα*$^{+/flox}$ and Cre negative tamoxifen-administered controls; and *Pdgfrα*$^{flox/flox}$ and Cre^{+ve} vehicle-administered controls (Figure S1C).

To further confirm appropriate excision of the targeted sequence, DNA was purified from the electrophoresis gel using a gel PCR-product DNA purification kit. A fragment of approximately 580bp was obtained after sequencing, containing a sequence upstream of exon 1, followed by part of the original targeting vector (NCBI GQ424832.1) (ref. 3), which contained the *loxP* site, followed by a fragment downstream of exon 4, confirming successful recombination and deletion of exons 1 to 4 from the *Pdgfrα* gene (see figure S1).

***Ex vivo* hair follicle formation assay**

We used the "patch" hair follicle formation assay [4,5] to assess the impact of PDGF on the ability of isolated hfDSCs to stimulate new hair follicle formation. Rat SKPs grown in the presence or absence of PDGF-AA and -BB were combined with epithelial aggregates extracted from backskin of newborn C57Bl/6 mice (P0; Charles River Laboratories). Dorsal skin was floated on 1mg/ml dispase for 30 min at 37°C in order to separate the dermis from the epidermis. The

dermis was discarded and the epidermis was transferred to a plate containing 0.25% trypsin (without EDTA) for 3-4 min at 37°C. To inactivate the enzyme, 10% FBS was added and then the epidermal sheet was gently scraped with a scalpel blade. Liberated epithelial cells were transferred to a 15 ml tube and centrifuged to 300g for 3 minutes and resuspended in DMEM. For each graft 10,000 epithelial aggregates were combined with 500,000 SKPs and subcutaneously injected under the back skin of adult male nude mice (Charles River Laboratories). After 14 days, grafts were harvested and the number of HFs containing GFP^{+ve} DPs within each graft were quantified (n = 3, with 4-6 grafts per treatment).

Preparation and administration of 4-hydroxy-tamoxifen (4-OHT)

For gavage administration, 100% ethanol was added to 25 mg of 4-OHT (H-7904, Sigma-Aldrich, St. Louis, USA) to obtain a 33 mg/ml 4-OHT suspension and aliquots containing 1 mg of 4-OHT were stored at -20°C protected from light until use. On the day of tamoxifen administration, 4-OHT solutions were warmed to 55°C before adding sterile sunflower oil (ethanol:oil; 1:9; v/v).

Immunofluorescence staining

To verify the location of PDGFRα positive cells within the adult dermal stem cell niche we examined anagen (P8, P28) or telogen (P56) backskin samples from PDGFRαH2BGFP mice (Jackson Laboratories). PDGFRαH2BGFP mice were created through knock-in of the histone H2Be-GFP fusion protein into the PDGFRα locus to allow for nuclear expression of GFP in cells that express PDGFRα [6]. Epithelial keratinocytes were labelled using keratin-5 (1:500; Covance). Cell death and cell proliferation was done with anti-cleaved caspase-3 (1:500, Millipore) and anti-Ki67 (Millipore). Dorsal rat skin or human skin samples were cryosectioned into 20 µm

slices, and mouse skin into 55 µm for lineage tracing experiments. To verify that adult human SKPs preserved their phenotype after exposure to the PDGF-B ligand, single cells were cytospun (Shandon) on slides at 800 rpm for 3 min and subsequently fixed in cold 4% PFA for 5 min. Cells were incubated with 10% normal goat serum containing 0.05% TritonX-100 for 1 hr and then exposed to primary antibodies against versican, αSMA, fibronectin and FSP1 overnight at 4°C. Tissue sections and cells were visualized with Alexa-488, -555 or -647 conjugated secondary antibodies (Life Technologies). Nuclei were labeled with Hoechst (1 µg/ml, Sigma Aldrich). Images were obtained using an Axioplan Observer microscope (Zeiss) or TCS SP8 spectral confocal microscope (Leica).

Real-time quantitative RT-PCR

mRNA was isolated from FACS-sorted cell populations using *αSMA*^{dsRed}*:Sox2*^{GFP} knock-in mice. RNA (27.6µg) was reverse transcribed using a High Capacity cDNA synthesis kit (Applied Biosystems). cDNA samples were amplified for 14 cycles using TaqMan® PreAmp Master Mix Kit (Applied Biosystems) and Taqman probes for *Pdgfrα*, *Pdgfrß* and the endogenous control, *Hprt* (Life Technologies, in either FAM or VIC formats). Preamplification was tested by comparing the ΔΔCT for each TaqMan® probe from a non-limiting sample of unsorted dermal cells amplified to the same sample that did not undergo amplification. qPCR was performed using TaqMan® Fast Advanced Master Mix and samples were run using 7500 Fast Real Time PCR system (Applied Biosystems). Fold-change in gene expression was calculated using the ΔΔCT method with *Hprt* serving as housekeeping gene. Cells from each hair follicle mesenchymal compartment (dermal sheath, dermal papilla and dermal cup) were compared to fibroblasts from interfollicular dermis. Probe details are shown below:

Hprt: ID: Mm03024075_m1 Chr.X: 52988078 - 53021660 on Build GRCm38

https://www.thermofisher.com/order/genome-database/details/gene-expression/Mm03024075_m1?pluginName=&CID=&ICID=#more-information-section

Pdgfr-α: ID: Mm00440701_m1 Chr.5: 75151401 - 75198211 on Build GRCm38

https://www.thermofisher.com/order/genome-database/details/gene-expression/Mm00440701_m1?pluginName=&CID=&ICID=

Pdgfr-ß ID: Mm00435546_m1 Chr.18: 61045150 - 61085068 on Build GRCm38

https://www.thermofisher.com/order/genome-database/details/gene-expression/Mm00435546_m1?pluginName=&CID=&ICID=

3. Supplemental references

1 Tallquist, M. D. & Soriano, P. Cell autonomous requirement for PDGFRalpha in populations of cranial and cardiac neural crest cells. *Development (Cambridge, England)* **130**, 507-518 (2003).

2 Wendling, O., Bornert, J. M., Chambon, P. & Metzger, D. Efficient temporally-controlled targeted mutagenesis in smooth muscle cells of the adult mouse. *Genesis (New York, N.Y. : 2000)* **47**, 14-18, doi:10.1002/dvg.20448 (2009).

3 Soriano, P. The PDGF alpha receptor is required for neural crest cell development and for normal patterning of the somites. *Development (Cambridge, England)* **124**, 2691-2700 (1997).

4 Biernaskie, J. *et al.* SKPs derive from hair follicle precursors and exhibit properties of adult dermal stem cells. *Cell stem cell* **5**, 610-623, doi:10.1016/j.stem.2009.10.019 (2009).

5 Hagner, A. & Biernaskie, J. Isolation and differentiation of hair follicle-derived dermal precursors. *Methods Mol Biol (Clifton, N.J.)* **989**, 247-263 (2013).

6 Hamilton, T. G., Klinghoffer, R. A., Corrin, P. D. & Soriano, P. Evolutionary divergence of platelet-derived growth factor alpha receptor signaling mechanisms. *Mol Cell Biol* **23**, 4013-4025 (2003).
