# Supplementary figures and images for: Platelet-derived growth factor signaling modulates adult hair follicle dermal stem cell maintenance and self-renewal
Source: NPJ Regen Med. 2017 Apr 14;2:11. doi: 10.1038/s41536-017-0013-4 (PMC5665619; doi:10.1038/s41536-017-0013-4)

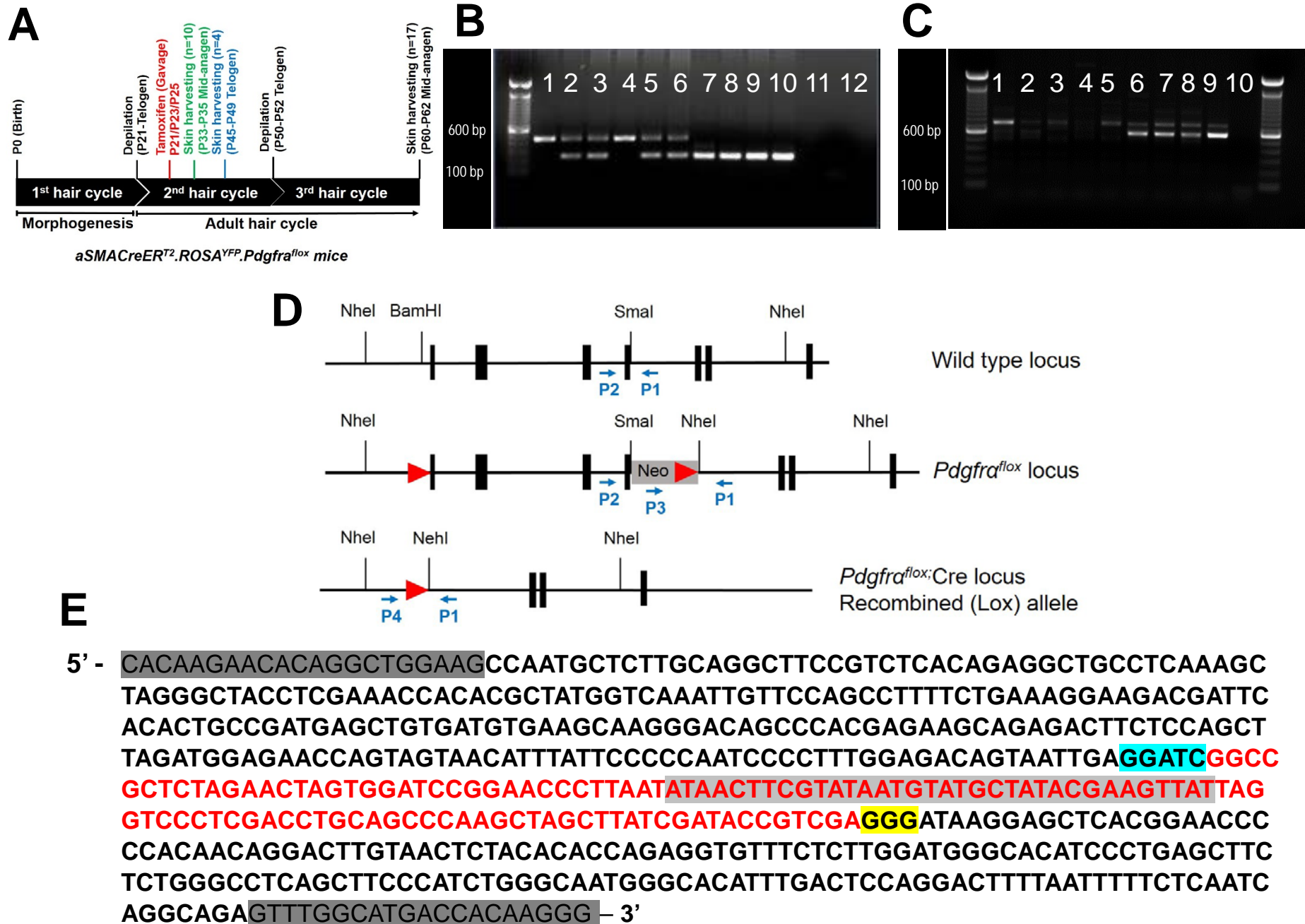

Figure S1

Supplement: Supplementary file 2 — Figure S1 [file 41536_2017_13_MOESM2_ESM.pdf]

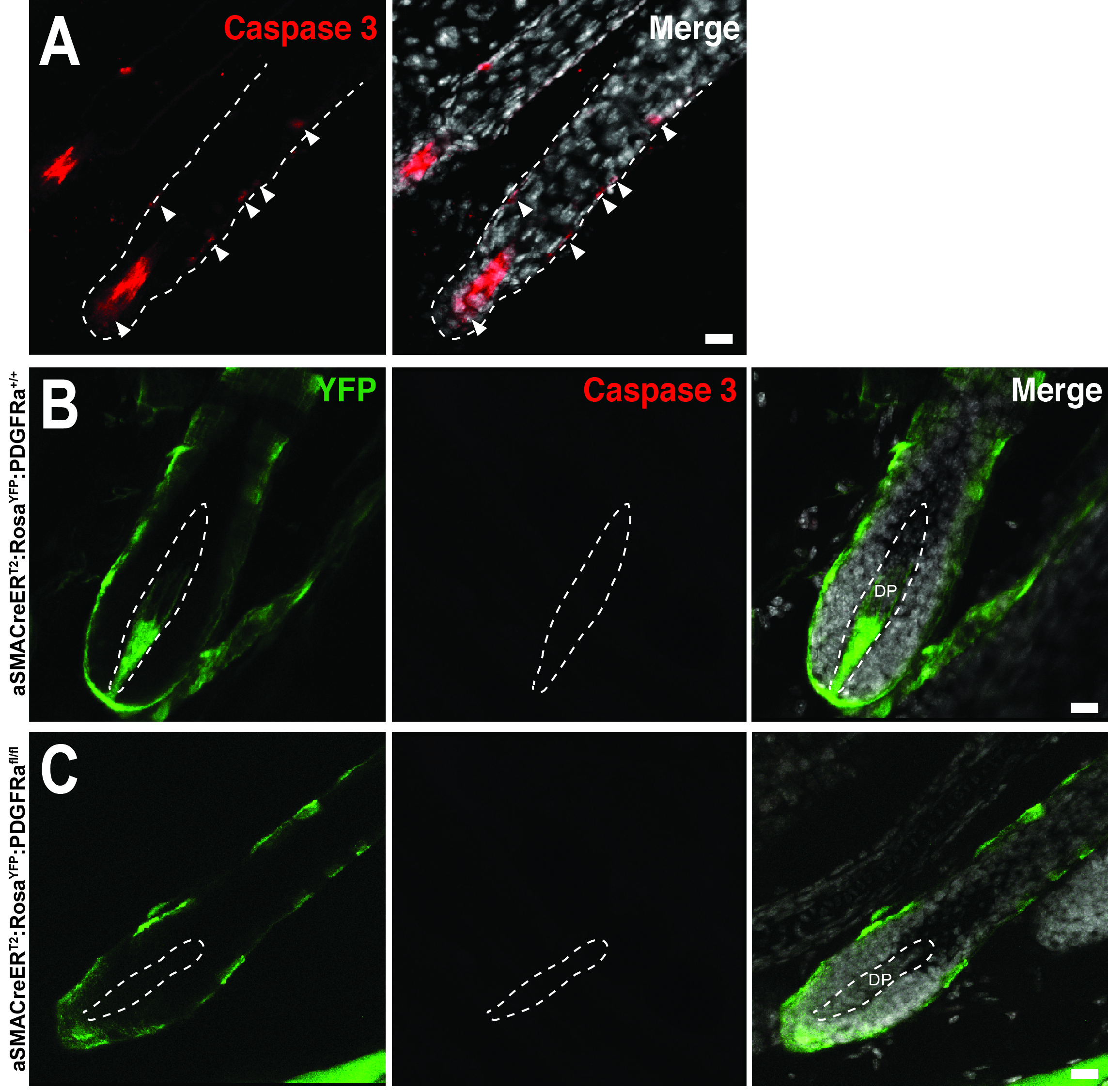

Supplement: Supplementary file 3 — Figure S2 [file 41536_2017_13_MOESM3_ESM.jpg]

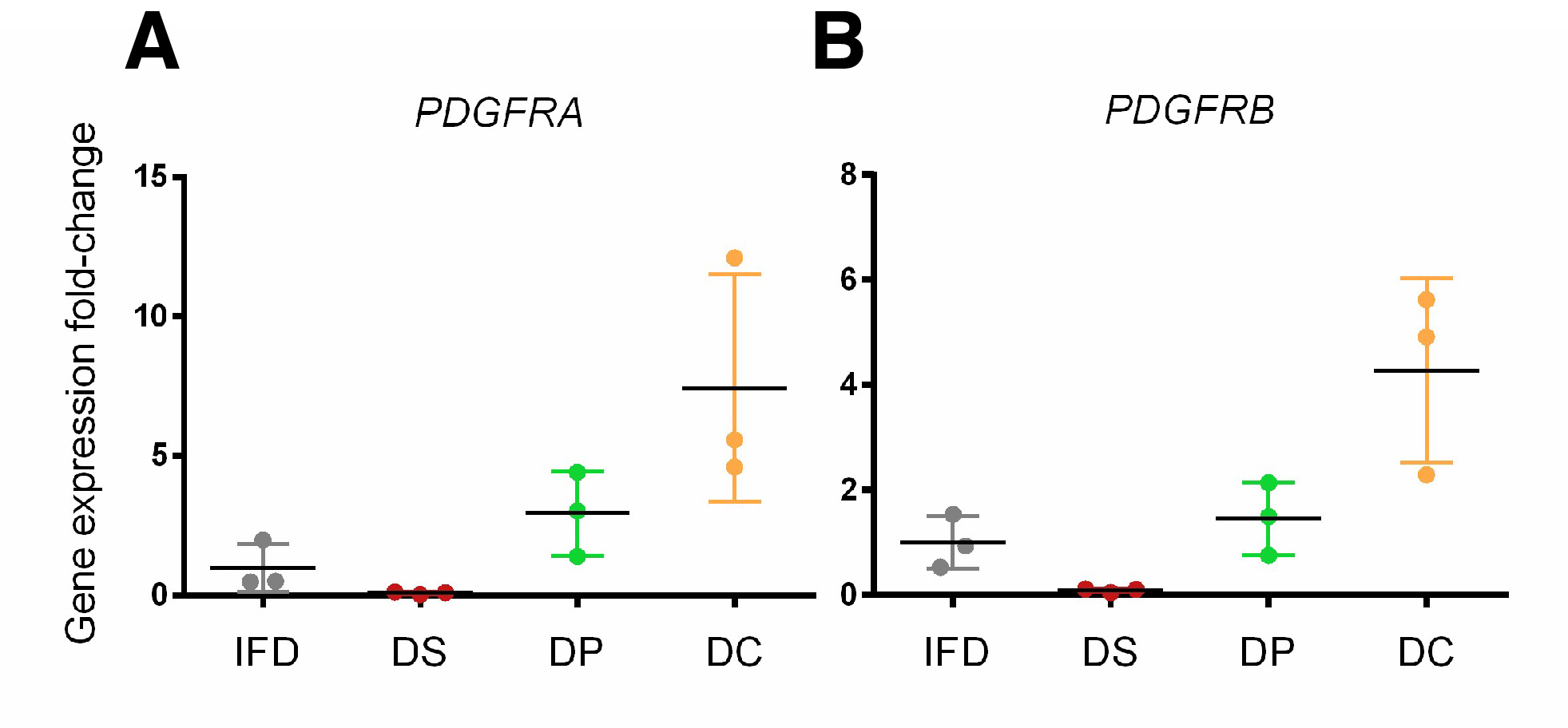

Supplement: Supplementary file 4 — Figure S3 [file 41536_2017_13_MOESM4_ESM.jpg]
